# Autonomous robotic surgery for zygomatic implant placement and immediately loaded implant-supported full-arch prosthesis: a preliminary research

**DOI:** 10.1186/s40729-023-00474-2

**Published:** 2023-05-19

**Authors:** Changjian Li, Menglin Wang, Huanze Deng, Shumao Li, Xinyu Fang, Yijie Liang, Xihua Ma, Yue Zhang, Yanfeng Li

**Affiliations:** 1 Department of Stomatology, The Fourth Medical Centre, Chinese PLA General Hospital, Beijing, 100048 China; 2 Medical School of Chinese PLA, Beijing, China; 3 Department of Orthopedics, The Fourth Medical Center of PLA General Hospital, Beijing, 100048 China

**Keywords:** Implant surgery, Robotic, Zygomatic implant, Immediate implant, Immediate restoration

## Abstract

**Objectives:**

A patient with extensive atrophy of the alveolar ridge in the posterior portion of the maxilla was selected to complete an experimental and clinical case of the robotic zygomatic implant to investigate the viability of an implant robotic system in clinical use.

**Methods:**

The preoperative digital information was collected, and the implantation position and personalized optimization marks needed for robot surgery were designed in advance in a repair-oriented way. The resin models and marks of the patient’s maxilla and mandible are all printed in 3D. Custom-made special precision drills and handpiece holders for robotic zygomatic implants were used to perform model experiments and compare the accuracy of the robotic zygomatic implant group (implant length = 52.5 mm, *n* = 10) with the alveolar implant group (implant length = 18 mm, *n* = 20). Based on the results of extraoral experiments, a clinical case of robotic surgery for zygomatic implant placement and immediate loading of implant-supported full arch prosthesis was carried out.

**Results:**

In the model experiment, the zygomatic implant group reported an entry point error of 0.78 ± 0.34 mm, an exit point error of 0.80 ± 0.25 mm, and an angle error of 1.33 ± 0.41degrees. In comparison, the alveolar implant group (control group) reported an entry point error of 0.81 ± 0.24 mm, an exit point error of 0.86 ± 0.32 mm, and an angle error of 1.71 ± 0.71 degrees. There was no significant difference between the two groups (*p* > 0.05). In clinical cases, the average entry point error of two zygomatic implants is 0.83 mm, the average exit point error is 1.10 mm and the angle error is 1.46 degrees.

**Conclusions:**

The preoperative planning and surgical procedures developed in this study provide enough accuracy for robotic zygomatic implant surgery, and the overall deviation is small, which is not affected by the lateral wall deviation of maxillary sinus.

## Introduction

For severely atrophied maxilla, zygomatic implant implantation is one of the effective repair method at present [1, 2]. The difficulty and complexity of zygomatic implant procedures, digital navigation systems, and surgery guides are occasionally used to assist surgeons with reasonable precision during the implant drilling and placement process [3–7]. However, in zygomatic implant surgery, the use of the designed digital guide is usually not fully realized, and there is considerable deviation in the use of soft tissue support guide, and it often fails in the insertion and fixation of bone support guide. In situations involving zygomatic implants, where the operating field is bigger, both the risk of guide insertion error and the probability of guide abandonment are increased. In addition, the guide plates suffer from poor operator visibility and necessitate a big mouth opening during operation, limiting their application in zygomatic implant surgeries. According to various studies, the deviation of dynamic navigation system assisted zygomatic implant surgery is less than that of guided assisted surgery [8]. The systematic analysis by Ramezanzade et al. [7] demonstrated that the dynamic navigation system is a viable technology for implant surgery assistance. However, at this stage, the pre-operation registration of dynamic navigation system, the installation and operation procedures of calibration equipment are complicated, and the safety and accuracy of this process largely depend on the experience and proficiency of surgeons. Consequently, the clinical implementation of the navigation system in zygomatic implant surgery remains challenging [9].

Since the advent of Da Vinci’s surgical robot system, many scholars began to apply robots to dentistry, including endodontics, orthodontics, oral and maxillofacial surgery, prosthodontics and dental implants. In 2004, Butscher et al. [10] invented an archwire bending robot named “SureSmile”, which can bend archwires into predetermined patterns more accurately and automatically. In 2010, Burgner et al. [11] successfully fabricated an orthognathic osteotomy robot based on a short-pulse laser ablation system. In 2014, Wang et al. [12] designed a miniature robotic device to offer a new method for crown preparation. Nowadays, implant robots are prevalent and well-established in clinical applications. They can better complete the placement of conventional implants (8–18 mm in length) at the alveolar ridge [13]. The benefits of robotic implants include precise positioning, minimally invasive surgery, reliable operation and repeatability.

The application of robot in clinical zygomatic implant surgery can theoretically improve the accuracy of the operation, but as far as the author knows, there are few reports on this. The reasons why robots have not been used in clinical zygomatic implant surgery may be as follows: (1) the limited operating space in the oral cavity necessitated a separately designed handpiece holder for the robotic zygomatic implant; (2) the long drill is more prone to deflection and slippage when drilling on the inclined surface of the zygomatic area and requires a specially custom-designed drill; and (3) zygomatic implantation surgery requires extensive flap turning, maxillary sinus opening and mucosal stripping, etc., and the existing marker will interfere with these procedures. If the existing marker of the robot system is installed in the patient’s mouth, it will interfere with the surgical operation and cause deviation, so it is necessary to redesign and improve the marker. The robot implantation accuracy of common implants can meet the clinical needs, but the research on zygomatic implants is not enough [7].

At present, robot-guided surgery can complete the implant implantation of patients with single tooth loss, multiple tooth loss and all-on-4 more effectively than traditional surgery. The robot system has achieved higher accuracy and the patient’s response is more comfortable. The purpose of this study is to conduct a model experiment and compare the differences between robot zygomatic implant (implant length = 52.5 mm) and alveolar ridge implant (implant length = 18 mm), so as to study the feasibility of robot application in zygomatic implant surgery, and report a case of robot zygomatic implant and full arch prosthesis supported by immediate loading implant, which was completed in a fully digital workflow approved by ethics.

## Materials and methods

### Initial status of the study subject and treatment plan

These data were obtained and used according to ethical requirements, and this study was approved by the Ethics Committee of the Fourth Medical Center of PLA General Hospital (approval number: 2022KY137-HS001).

A 45-year-old male patient, ASA level 1, had no previous history of systemic diseases, such as diabetes, osteoporosis or immunodeficiency, but his legs were limited, and volunteered to be the research subject. The patient came to the hospital because of loose teeth in the upper and lower jaws for more than 3 years, accompanied by bleeding gums and weakness in chewing, and asked for comprehensive oral rehabilitation to restore normal chewing function and beautiful teeth. The patient denied smoking or a specific family history. The patient’s oral and informed consent was obtained using the relevant information.

**Fig. 1:**
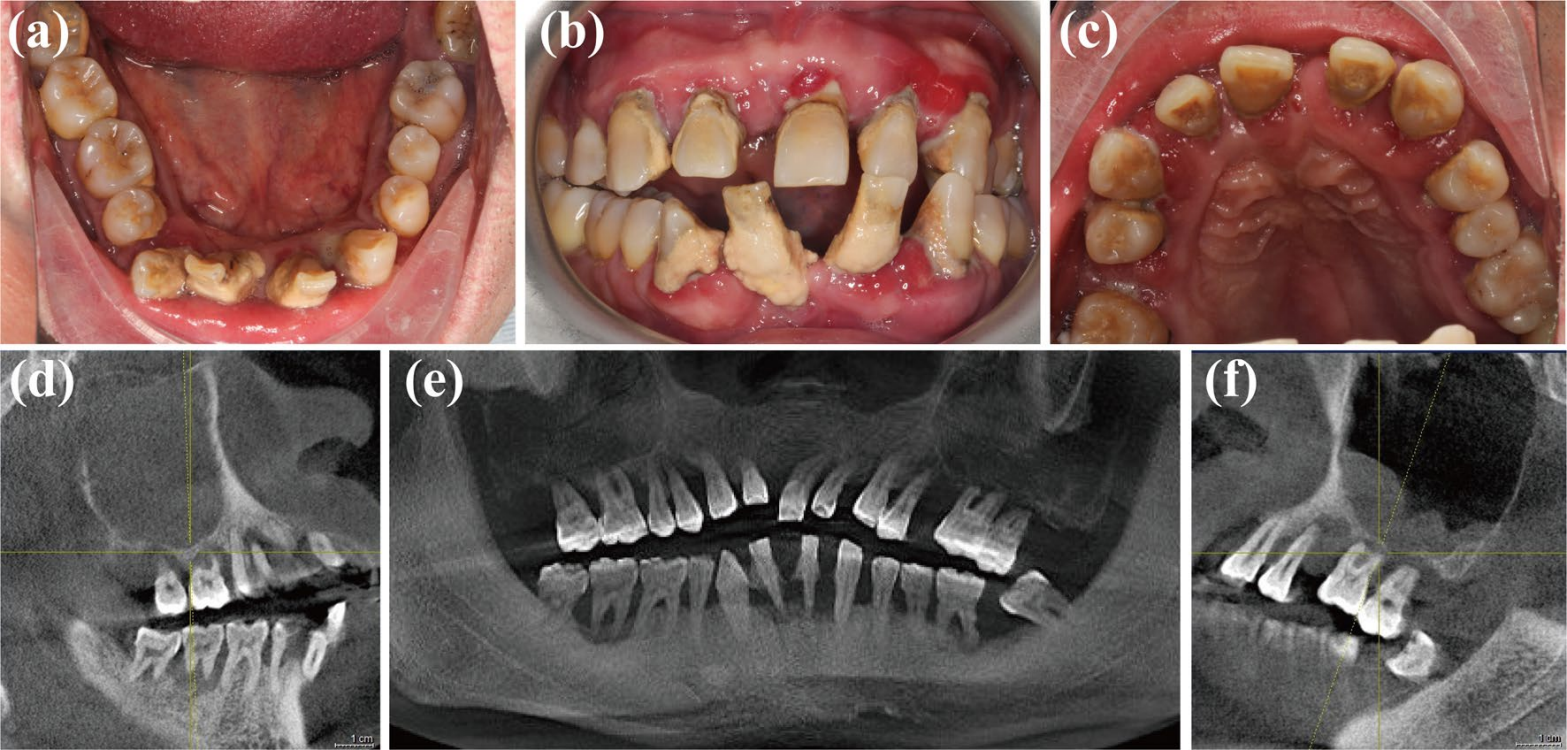
Initial dental status of the patient. The initial intraoral examination showed poor oral hygiene and severe periodontitis with missing teeth (**a**–**c**). The panoramic radiograph showed obvious bone deficiency (**e**) and inflammation of bilateral maxillary sinus, with the right side more severe than the left side (**d**, **f**)

The extraoral examination revealed no obvious abnormalities. No clicking or pain was noted in the temporomandibular joint region. Initial intraoral examination showed poor oral hygiene, PLI = 2–3, CI = 1–3 (Fig. 1a–c). The gingival tissue was red and brittle, and the gingival papillae were hypertrophied, rounded, and detached from the tooth surface, BI = 3. Gingival recession was observed in the anterior region from the cervical 1/3 to the middle 1/3 of the root. Pd = 5–10 mm, Al = 3–9 mm. Most teeth show II–III activity. The front teeth are displaced in a fan-shaped labial direction. The #11/25/31/37/42 (the symbol of the World Dental Federation of Foreign Direct Investment) is missing, and scattered gaps can be seen in the front area. The #36/46 tooth showed superficial buccal fissure caries with class I activity. A deep overbite and unstable posterior occlusion were detected during central occlusion, and anterior guidance was missing during mandibular movement. Cone-beam computed tomography (CBCT, Orthophos XG, Dentsply Sirona, German) examination showed most alveolar bone resorption extended to the apical 1/3 on both maxilla and mandibula (Fig. 1e). Severe bone deficiency and bone height deficiency were detected in bilateral posterior regions. Inflammation occurred in both maxillary sinuses, and the right side was more serious than the left side (Fig. 1d, f).

According to the requirements of patients, the prosthesis supported by full arch fixed implants was recommended to patients. The patient consented to the use of an autonomous robotic oral surgery system (Remebot, Beijing Rui Yi Bo Technology Co., Ltd.; Beijing, China) to perform immediately loaded full-arch fixed implant rehabilitation on the edentulous maxilla and mandibula, with two distal zygomatic implants on the maxilla.

### Preoperative planning

**Fig. 2:**
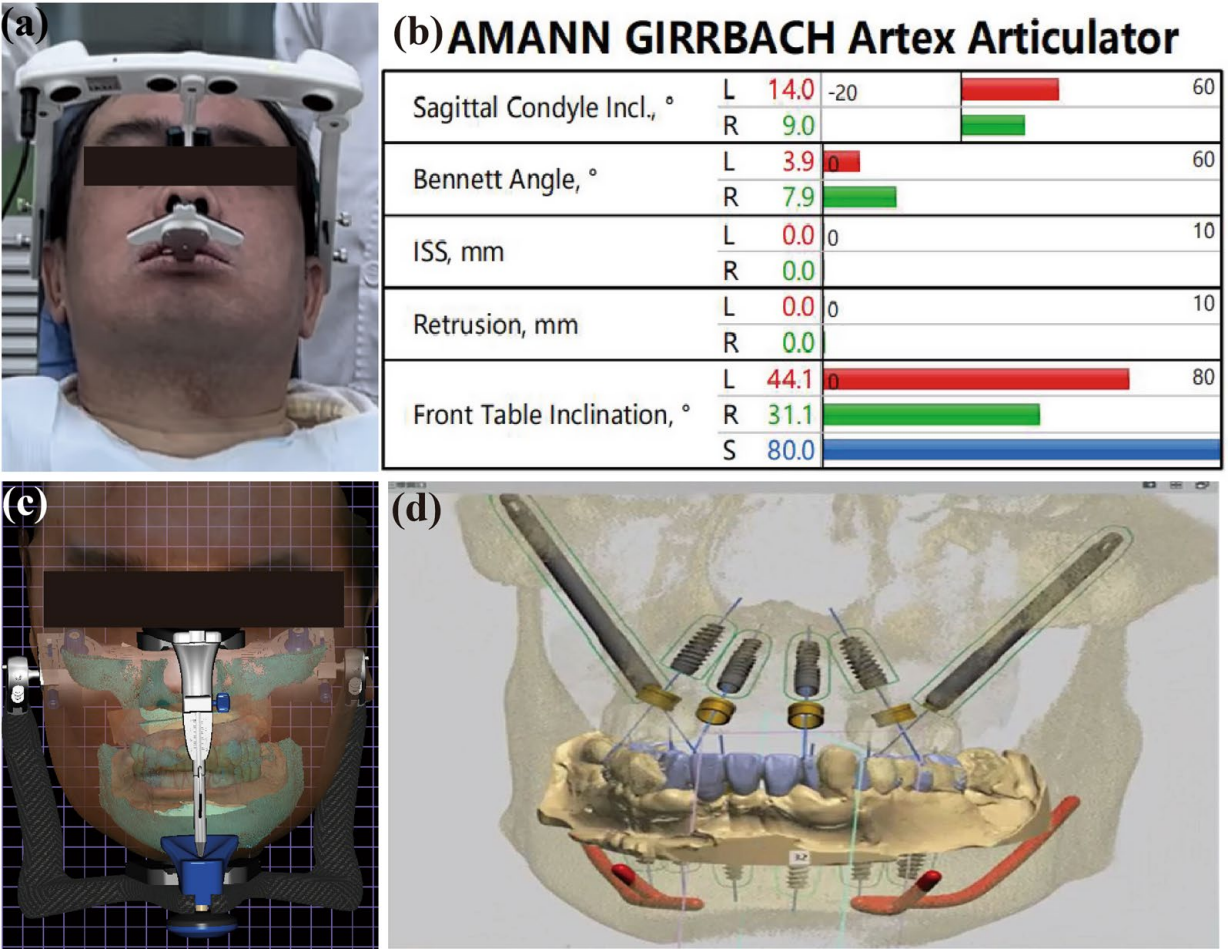
Patient wearing an electronic face bow for collection (**a**); electronic face bow data (**b**); data fusion of oral scan, face scan, CT and other data, and virtual combined tooth arrangement (**c**); restoration-oriented planting planning (**d**)

**Fig. 3:**
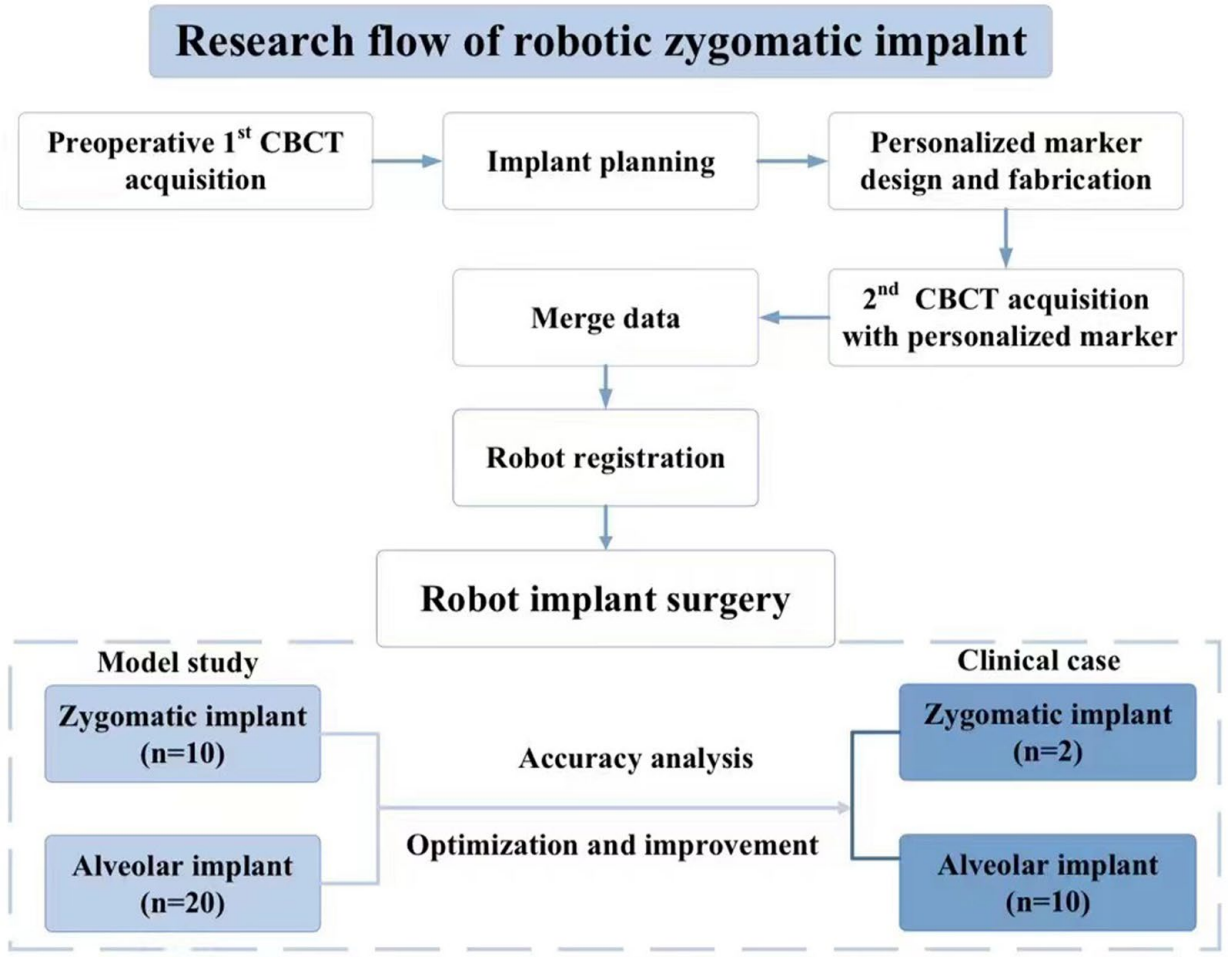
Research flow of robotic zygomatic implant

Robotic surgery was planned through an innovative complete digital workflow. A variety of digital devices including intraoral scan (iTero, Align, USA), the 3D facial scanner (Obiscanner, Wecare Digital, China), the artificial facebow (JMA Optic, Zebris Gmbh, Germany) were used to collect preoperative information of the patient and to develop a personalized plan for the implant surgery and immediate restoration (Fig. 2a, b). Integrate the data into the computer and design a fixed implant prosthesis on the virtual articulator (Fig. 2c). With regard to the relationship between the soft tissue morphology and the position of maxilla, the virtual position relationship between maxilla and mandible teeth was determined. CBCT data of patients were imported into robot implant planning software (RemebotDent, Remebot, Beijing Rui Yi Bo Technology DICOM, Ltd.; China), and the digital information collected before operation. Finally, the robot system determined the position of the implant based on a restorative-oriented methodology to complete the implant surgery planning (Fig. 2d). The research flow of the robot-assisted implant surgical system is shown in Fig. 3.

### Design of the marker for robotic zygomatic implant surgery

**Fig. 4:**
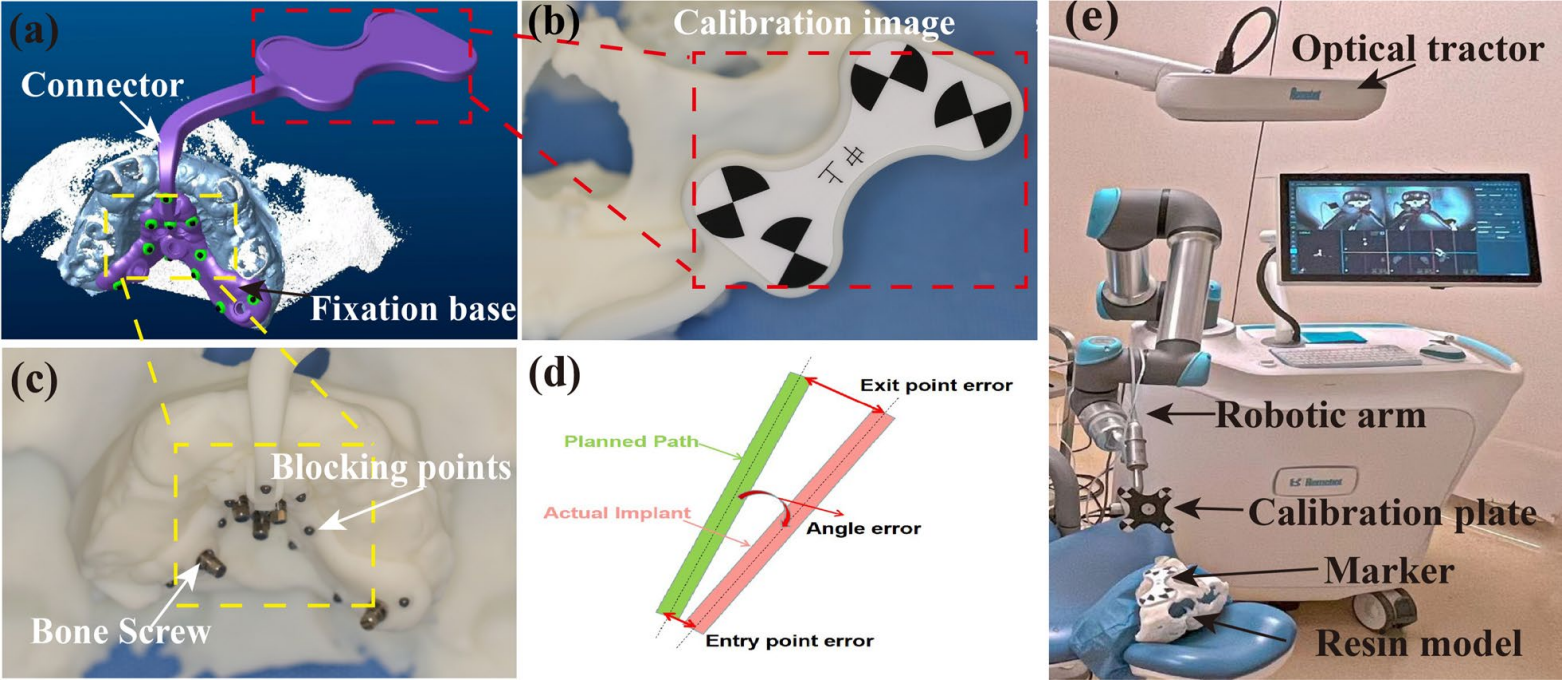
Design personalized marker (**a**); fix the marker on the resin model (**b**, **c**); schematic diagram of implant deviation (**d**); robot arm and marker registration (**e**)

The positioning guide used in the robotic registration process (the marker) was personalized-designed in this study (Fig. 4a, b). The marker was optimized to reduce the size and make it more stable without affecting the surgical procedures. The marker was designed to be fixed in a region with greater bone quantity on the maxilla by bone screws. The fixation base with blocking points for localization was designed in a herringbone shape for the more contacting area to gain more stability (Fig. 4c). The distribution of blocking points on abutment is designed to be staggered in height and close to the edentulous area to obtain higher accuracy. The fixed base and the marker are connected by a narrow and thick connector, which has a cross section of 7.00 mm × 4.50 mm in the mouth and gradually becomes 7.00 mm × 6.00 mm after leaving the mouth (Fig. 4a). The connector avoids the front area (where the implant is designed) and the upper lip, and has a certain buffer space. The marker was made of hard resin (Surgical Guide UV, HEYGEARS, Guangzhou, China) which was not easily deformed. The calibration image plate of the marker was designed in black-and-white blocks with ambient light recognition technology (Fig. 4b), positioned to avoid the operative area as much as possible, and was not easily obscured by the operator.

### Extraoral experiment of robotic zygomatic implant placement

Five sets of resin (Somos EvoLVe 128, DSM, Netherlands) models of the patient’s upper and lower jaws were printed with a 3D printer (Matrix 520, UnionTech, China). Fix the marker on the resin model with bone screws, take out the second CBCT and introduce it into the system. The marked data are merged with the first CBCT data to confirm the 3D position where the implant is expected to be placed.

The implant handpiece was attached to the bespoke metal holder at the end of the robotic arm, and then a calibration plate with black-and-white positioning blocks was attached to the handpiece where the drill was installed during the surgery. Start the robot, and register the robot arm with the CBCT data with the marker (Fig. 4e). The mechanical arm moves in six positions around the resin model in turn (these six positions cannot be coplanar and should include the implantation area). The coordinates of the calibration plate in the space of the optical tracking positioner and the manipulator are recorded, respectively. Through coordinate transformation, the spatial registration of the manipulator and the optical tracking position is completed. In this process, the positioning calibration board on the marker also receives the coordinates, which allows the spatial registration between CBCT data and the robot arm to be realized by extracting the coordinates of the marker in the space of the optical tracking locator and the robot arm, respectively, and through transformation and calculation in the robot system.

**Fig. 5:**
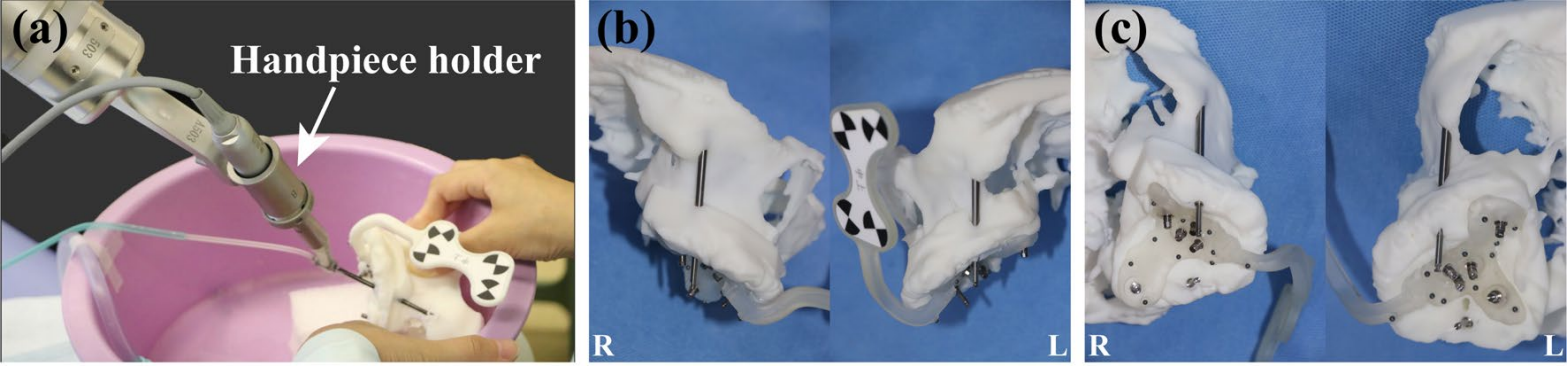
In vitro model study was conducted on the 3D printed resin maxillary and mandibular model before clinical surgery (**a**). The frontal view (**b**) and the lateral and posterior view (**c**) of robotic zygomatic implant on resin models exhibited satisfying position and orientation

The extraoral experiment was conducted to verify the accuracy of robotic zygomatic implant (52.5 mm, 45°NobelZygoma, Nobel Biocare, Swedish) placement compared to normal implant (4.3 × 18 mm, NobelActive RP, Nobel Biocare, Swedish) placement on the model. The experimental group consisted of 10 zygomatic implant sites on the left and right sides of 3D-printed models, while the control group consisted of 20 common implant sites on the alveolar crest of the models. The model was placed in a position simulating the patient’s position, and surgical drilling and implantation were performed under the same conditions (Fig. 5a). During the operation, the mechanical arm can move synchronously with the tiny movement of the model and the patient’s head. In the experiment group’s site preparation, the following order of drills was utilized (Fig. 12a): precision drill 2.0 × 33 mm, twist drill 2.0 × 31 mm, twist step drill (2.4/2.8 × 31 mm, 2.8/3.2 × 31 mm, 3.2/3.6 × 31 mm), precision drill 2.0 × 74.8 mm, twist drill 3.0 × 67.5 mm, pilot drill 3.5 × 75 mm, and finally twist drill 3.5 × 67.5 mm. In the control group, site preparations were achieved under the instruction of the manufacturer.

### Accuracy analysis

After sites preparation and implant placement, the postoperative CBCT was taken out and input into the robot system, and integrated with the preoperative CBCT, and the 3D position of the implant was planned before operation. The error of entry point, exit point and angle error between actual position and planned position are calculated to check the accuracy of robot implant placement [14] (Fig. 4d). The entry point error represents the change of cervical spine center between the actual implantation position and the expected implantation position. The exit point error represents the difference between the actual position and the planned position of the apex center. The angle error represents the difference between the actual long axis of the implant and the long axis of the implant planned before operation. The results were measured and recorded by the same experienced inspector. After performing the normality test and chi-square analysis, the *t* test for independent samples was utilized to determine the statistical difference between the two groups. Significance was considered at *p* < 0.05.

## Results

In the experimental group involving in-vitro robot zygomatic implant surgery, the average entry point error is 0.78 ± 0.34 mm, the average vertex error is 0.80 ± 0.25 mm, and the average angle error is 1.33 ± 0.41 degrees (Fig. 5b, c). Meanwhile, in the control group for conventional robotic alveolar implant placement, the mean entry error was 0.81 ± 0.24 mm, the mean exit point error was 0.86 ± 0.32 mm, and the mean angle error was 1.71 ± 0.71 degrees. No significant difference was detected between the two groups with *p* > 0.05 (Table 1).

**Table 1:** Accuracy parameters of the experimental group and the control group (means ± standard)

Group (*n*)	Entry point error (mm)	Exit point error (mm)	Angle error (°)
Experimental group (*n* = 10)	0.78 ± 0.34	0.80 ± 0.25	1.33 ± 0.41
Control group (*n* = 20)	0.81 ± 0.24	0.86 ± 0.32	1.71 ± 0.71
*P* value	0.74	0.62	0.14

## Case study

### Preoperative treatment

**Fig. 6:**
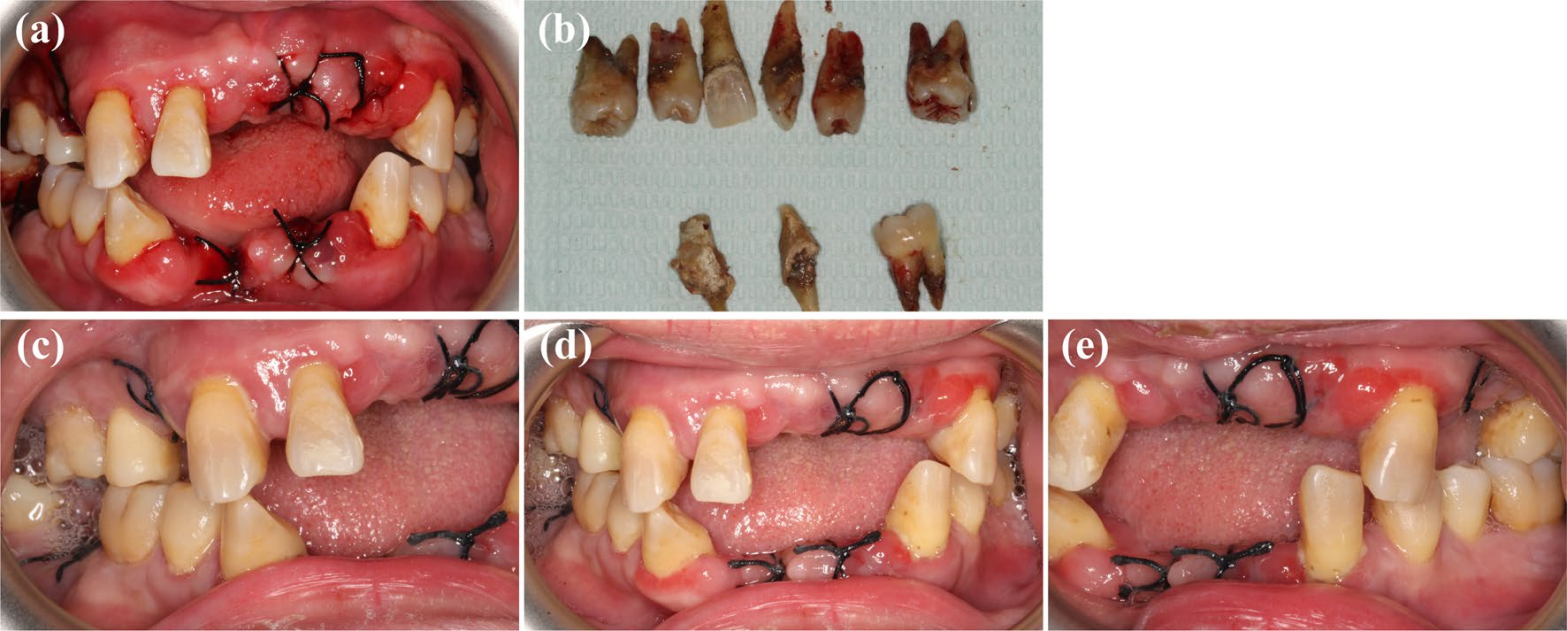
Preoperative periodontal was given to the patient and severely loosen teeth with obvious infection (**b**) were extracted in a minimally invasive way (**a**). One week after extraction and periodontal treatment, the gingival swelling had subsided (**c**–**e**). Oral hygiene maintenance was emphasized to the patient for the following treatment

The initial intraoral status and the treatment plan were previously described. The patient’s maxillary sinusitis was primarily attributable to an odontogenic infection, according to the opinions of the ENT department. Before implantation, basic periodontal treatment was given in the stage of acute symptom control. Painless periodontal scaling under local anesthesia and minimally invasive extraction of severely loose teeth were performed at the same time (Fig. 6a, b). The patient’s odontogenic infection was extracted and treated with antibiotic combination for 1 week. Through the painless and comfortable periodontal treatment stage, patients gained trust in the follow-up treatment and their compliance was enhanced. One week after extraction, sutures were removed and the gingival swelling had subsided (Fig. 6c–e), maxillary sinus infection and nasal congestion progressively restored to normal. During this time, the patient maintained good dental hygiene for the next 3 months of the periodontal recovery period.

### Surgery procedure

**Fig. 7:**
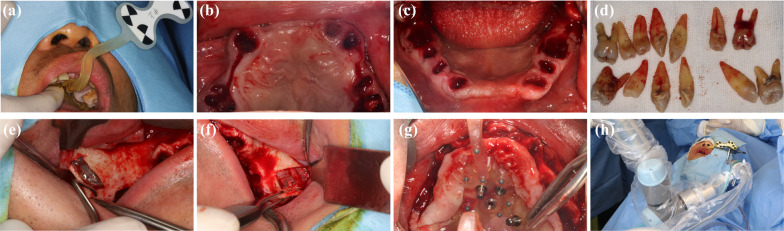
Intraoperative phase of robotic implant. The marker was tried on prior to the beginning of procedure (**a**). The residual teeth on maxilla (**b**) and mandibular (**c**) were extracted as previously planned (**d**). Flap opening (**e**), maxillary sinus wall opening (**f**), and the mucosa stripping were achieved before fixation of the marker with bone screws (**g**). The robotic system and the marker were registered for autonomous operation of the robotic arm (**h**)

Using the above combined data, personalized marks are constructed, printed, tried on, and attached to soft tissues with good stability before disinfection for surgery. Before the operation, the maxillary nerve and infraorbital nerve were blocked and anesthetized. Sterilize the operation area before placing the sterile operation sheet. The modified positioning marker was tried on to ensure its stability (Fig. 7a). Residual loosen teeth were extracted in a minimally invasive way except for #36/46 (Fig. 7b–d).

During surgery, the gingival mucosa of the maxillary vestibular sulcus from #17 to #27 was incised and flipped, the lateral wall of the bilateral maxillary sinus was opened (Fig. 7e, f), and the mucosa of the maxillary sinus near the path of the zygomatic implant was peeled off. The marker was fixed in the set position by bone screws (Fig. 7g). The patient was examined by CBCT with the marker in place. The 3D position information of the implant planned before operation is fused with the marked CBCT image during operation, and the planned position of the implant and the mark is confirmed again. The registration between the positioning marker and the robotic arm was performed afterward (Fig. 7h).

**Fig. 8:**
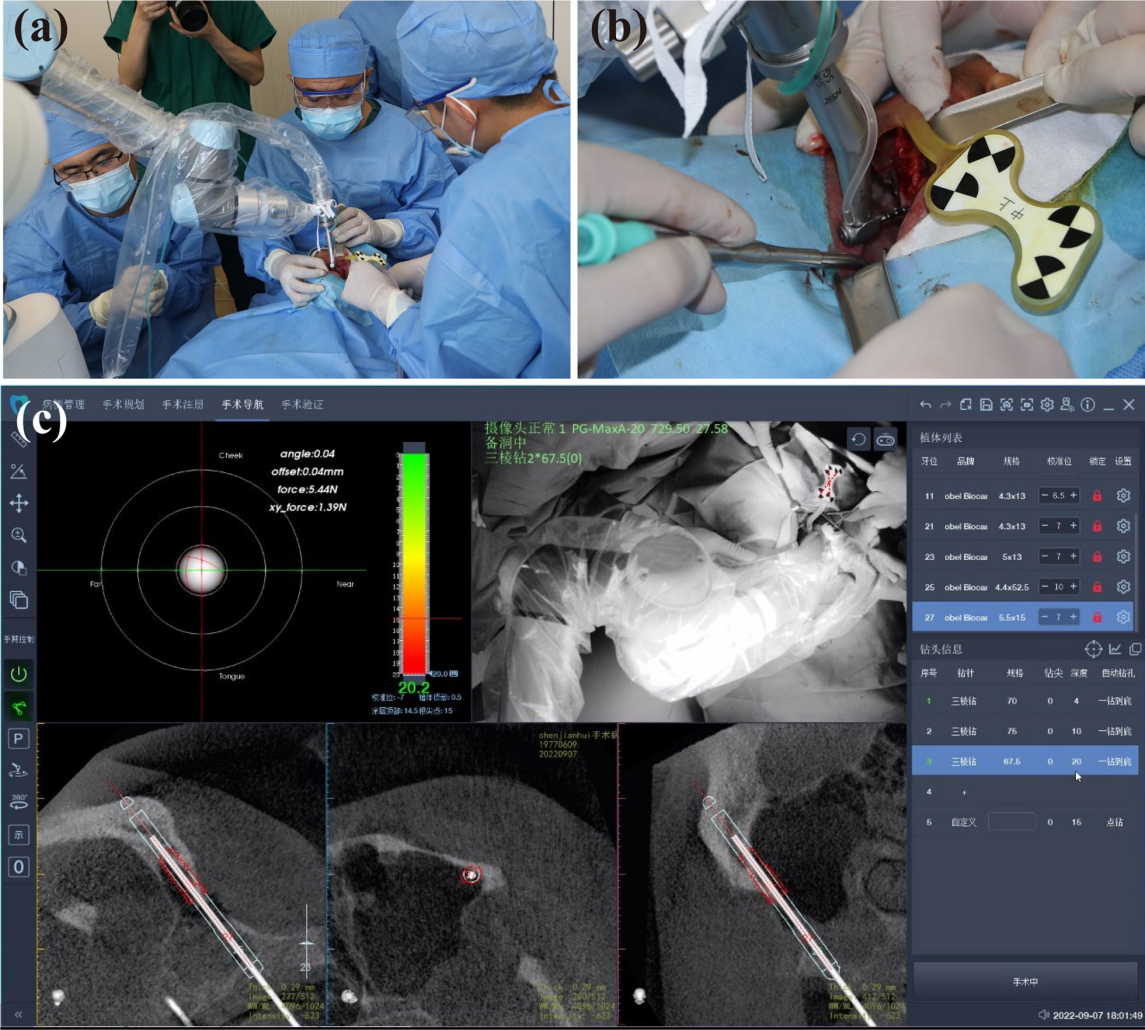
Intraoperative image and autonomous site preparation procedure. The autonomous zygomatic implant site preparation was achieved by the image-guided robotic surgery system (**a**, **b**). Robot-assisted surgical software system provide real-time feedback of the robotic arm and the drill in surgery (**c**)

**Fig. 9:**
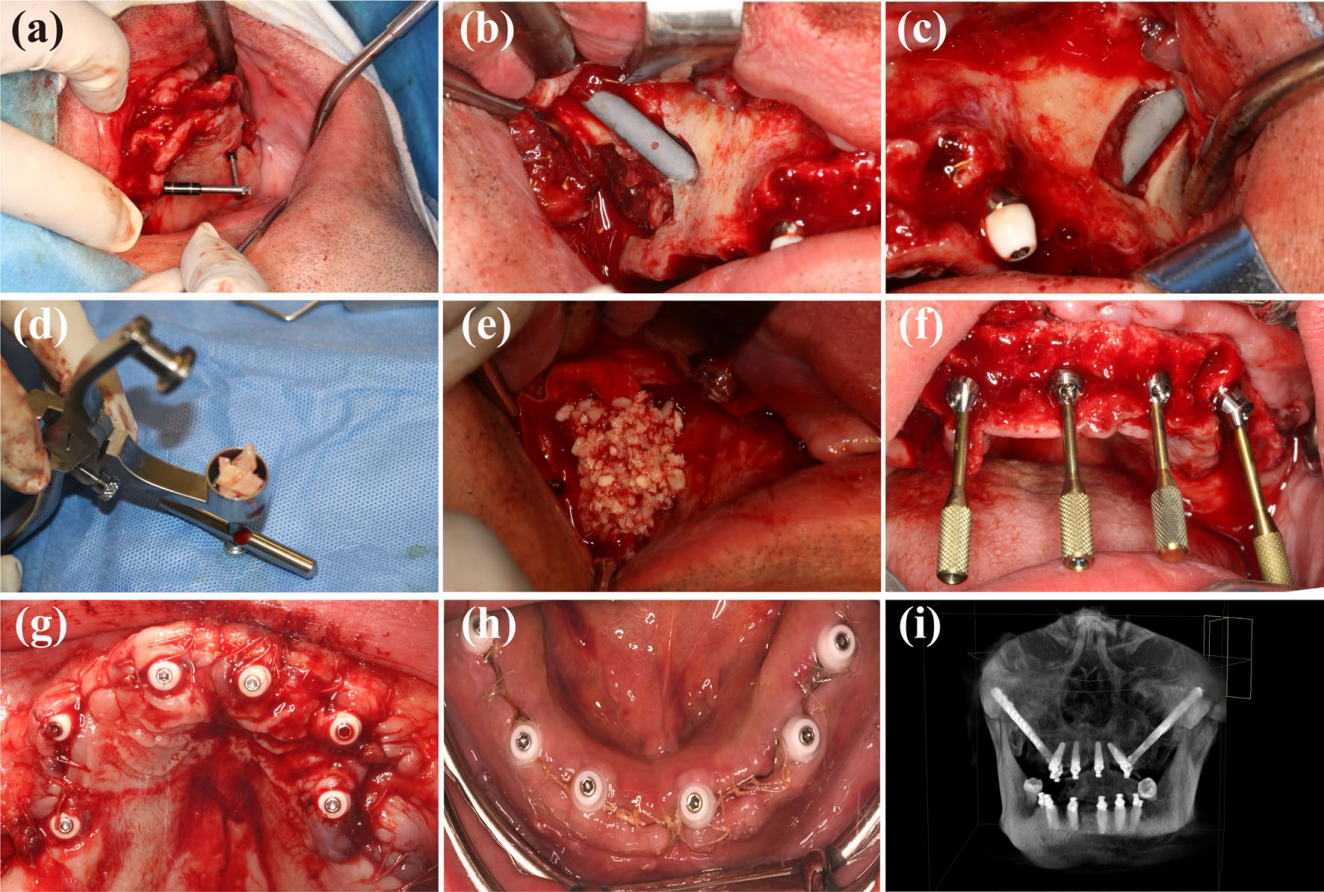
After sequential preparation for the zygomatic implant, the light-blocking long drill was inserted (**a**) and the intraoperative CBCT was taken. After confirmation of the orientation and the depth of site preparation, bilateral zygomatic implants were placed (**b**, **c**). Autologous bone was collected and grounded (**d**) and grafted into bone deficient area (**e**) together with Bio-Oss (Geistlitch, Pharma AG, Wolhusen, Switzerland). Other alveolar implant placement was completed with robotic assistance and composite abutments and healing caps were placed (**f**–**h**). Postoperative CBCT was taken (**i**)

According to the preoperative plan, the manipulator automatically determines the position and direction of the implant (Fig. 8a, b). Through the screen of the robot system, the surgeon can see the operation method and many indexes in real time from all directions (Fig. 8c), and they can control the movement of the manipulator at any time with the pedal. When the lateral deviation of the field preparation process exceeds 0.5 mm, the software will sound an alarm. When the drill reaches the required depth, the system will send a signal, and the mechanical arm will automatically leave the site and reset to its calibration position. “Extraoral experiment of robotic zygomatic implant placement” section describes the drill sequence during site preparation. After zygomatic sites preparation, the drills were inserted and the intraoperative CBCT was taken to confirm the direction (Fig. 9a). The orientation was satisfying, and the preparation of the site continued to 3.5 × 68 mm. The 52.5 mm length zygomatic implants were placed on both sides, and the initial torque of 35 N was confirmed. The 0-degree straight composite abutment was screwed into the site of #16 meanwhile the 17-degree angled composite abutment was screwed into the site of #26 (Fig. 9b, c). For the site of bone defect, the collected autogenous bone was ground and mixed with Bio-Oss (Geistlitch, Pharma AG, Wolhusen, Switzerland), and then transplanted into the defect area (Fig. 9d, e). The bone graft area was covered with Bio-Gide membrane (Geistlitch, Pharma AG, Wolhusen, Switzerland), and the soft tissue was sutured with reduced tension. Other implants were placed with the help of robots, and composite abutments and healing caps were placed (Fig. 9f–h).

### Postoperative procedure

**Fig. 10:**
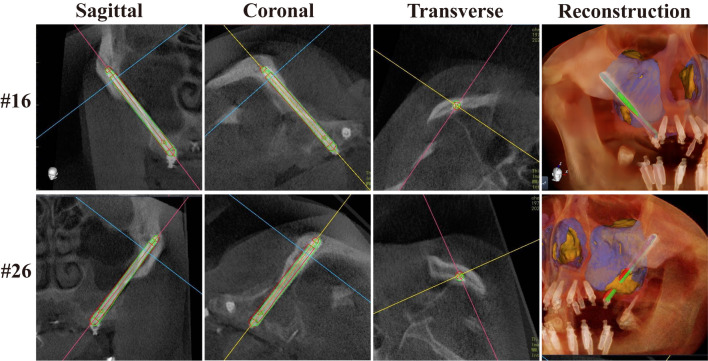
Accuracy analysis for deviations between the planned and placed zygomatic implants in sagittal plane, coronal plane, transverse plane and the reconstructed images. The planned implant was represented by the red profile and the actual implant was represented by the green profile

**Fig. 11:**
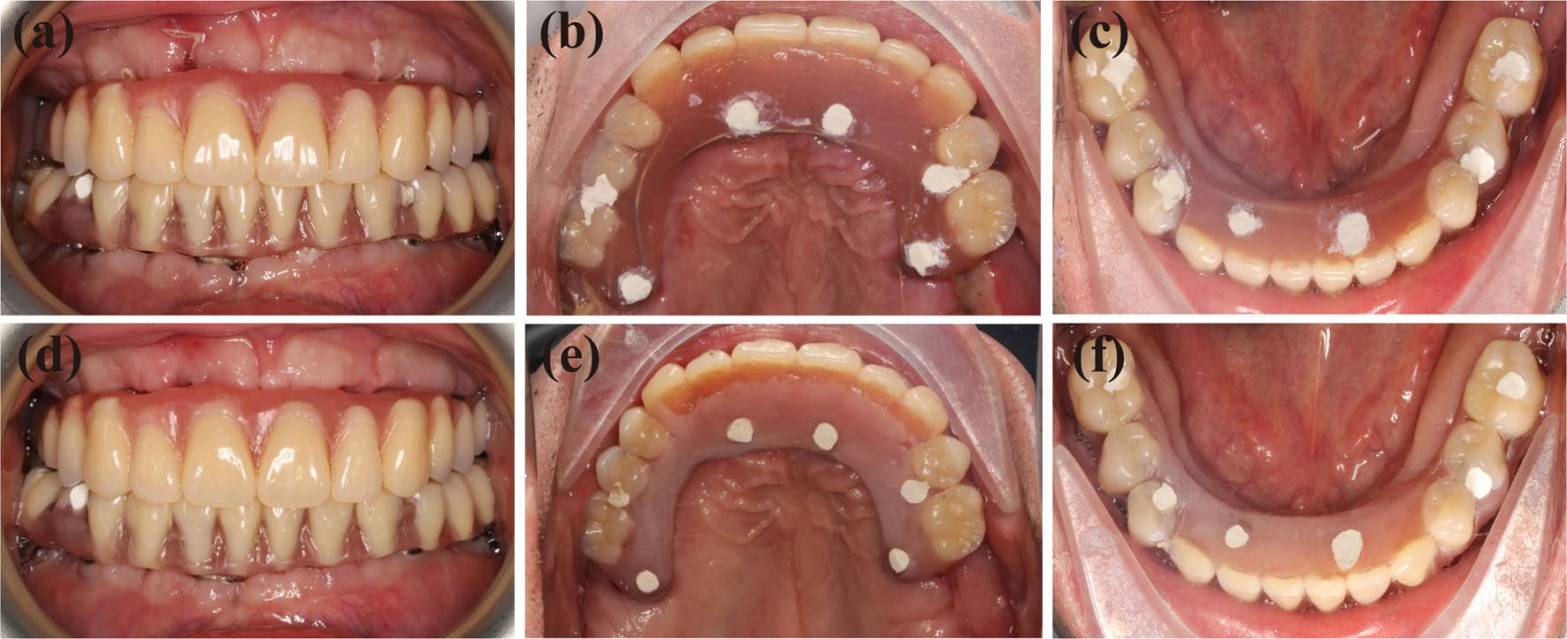
Immediate loaded implant-supported full arch prosthesis was given to patient right after the surgery (**a**–**c**). At the 3-month follow-up visit, the patient showed acceptable intraoral conditions and was satisfied with the aesthetic appearance and the restored function brought by the prosthesis (**d**–**f**)

No adverse surgical event was reported during the use of the robotic oral surgery system. The patient underwent postoperative CBCT examination after all implants on both arches were placed (Fig. 9i). Based on integrated data, the placements of the actual zygomatic implant and the designed implant were compared (Fig. 10). The errors of entrance point and exit point of #16 are 0.98 mm and 1.02 mm, and the angle error is 1.21 degrees. The errors of entrance point and exit point of #26 are 0.68 mm and 1.17 mm, and the angle error is 1.70 degrees. Immediate prosthodontics is used for total prosthodontics, and self-oral hygiene maintenance is emphasized (Fig. 11a–c). The patient was satisfied with the immediate restoration and was instructed to pay a follow-up visit in 3 months. At the 3-month follow-up visit, the patient presented with acceptable oral hygiene and periodontal condition (Fig. 11d, e). The patient was satisfied with the aesthetic appearance and the restored chewing function and reported no unpleasant feelings.

## Discussion

In 1996, the Da Vinci Surgical Robot System (Intuitive Surgical, California, USA) was developed, and surgical robots have achieved great success in the medical field by making complex surgeries safer and less invasive [15]. With the development of surgical navigation technology, dental implant surgery robots are becoming more and more popular, and have been successfully applied to clinical patients so far.

In 2017, Yomi, the world’s first commercial dental robot, was developed [16, 17]. According to whether the manipulator needs the manual control of the surgeon, Yomi is a semi-automatic implantation robot, which can provide physical guidance through touch, vision and hearing, and needs the manual control of the surgeon during the implantation process. In 2021, Bolding et al. [13] used Yomi for the placement of 38 implants and reported an angle error of 2.56 ± 1.48 degrees, an entry point error of 1.04 ± 0.70 mm, and an exit point error of 0.95 ± 0.73 mm. Zhao et al. [18] introduced the world’s first autonomous dental implant placement system (Yakebot, Beijing, China) at the end of 2017 and ushered in the digital era of dental implantation. In 2021 animal experiment the experimental results showed that the error of entry point was 0.27 ± 0.15 mm, the error of exit point was 0.25 ± 0.22 mm, and the angle error was 0.99 ± 0.52 degrees [19]. The system is proved to be more accurate than the digital whole process guide. At the beginning of 2021, China National Medical Products Administration authorized two autonomous dental implant robot systems, Remebot (registration number 20213010713) and Yakebot (registration number 20213010215). Remebot and Yakebot are both highly autonomous robotic systems; their robotic arms can execute intraoral implant surgery duties directly with follow-me functionality, without the need for human intervention. In 2022, Yang et al. [15] used Remebot to complete a case of the edentulous maxilla with an entry point error of 0.56 ± 0.24 mm, an exit point error of 0.61 ± 0.23 mm, and an angle error of 0.99 ± 0.52 degrees.

In recent years, the application and research of robots in complex cases have made steady progress. In the case of massive bone loss in maxillary posterior teeth, zygomatic implant is one of the best treatment methods for edentulous restoration. maxillary sinus external lift procedure, the zygomatic implant could allow for an immediate restoration. The aforementioned robotic systems have been focusing on the placement of common implants less than 18 mm on the alveolar crest and further research is needed on the precision and safety of robotic placement of extra-long implants (30–55 mm) in unique structural locations.

At present, there are limited experimental and clinical studies related to robotic zygomatic implant surgery. In 2019, Cao et al. [20] developed a 6 DOF zygomatic implant robotic system. In vitro experiments revealed that the entry point error, the exit point error, and the angle error of this robotic system were 0.79 ± 0.19 mm, 1.49 ± 0.48 mm, and 1.52 ± 0.58 degrees. The robot system in this study may have the following limitations, which prevent it from being implemented in clinic: (1) the implantation handpiece used in this system is close to the straight handpiece with minimum angular flexibility. When the implantation site is in the rear area or when the patient’s mouth opening is insufficient, the robot system may not be able to enter the oral cavity; (2) the mark of its robot system is fixed on the skull, and it is invasive with clinical secondary trauma; (3) the blocking point located by this system is the bone screw fixed on the anterior alveolar ridge, which is also traumatic and inaccurate; (4) its headstock is made of resin material, which is large in size and may be deformed when subjected to high lateral force; (5) the robot system lacks the feedback function of the lateral force of the drill bit, which leads to a large deviation in the use of the long drill bit (65–100 mm) subjected to the lateral force during the preparation of the zygomatic implant site; and (6) the extraoral experimental model cannot completely simulate the clinical environment of patients with insufficient information of soft and hard tissues.

At present, the preoperative imaging examination of medical CT or more advanced cone-beam CT can well-evaluate the bone, bone mass, anatomical shape and surrounding anatomical structure of the implant area. CBCT has a small amount of radiation, and its size measurement can reach an accuracy of 0.1 mm, which is equivalent to the level of multi-slice computed tomography (MSCT) [21]. It is generally believed that there is no difference between the measured data of craniomaxillofacial region in CBCT imaging in clinical application and the actual measured data, which can accurately and truly display the anatomical structure [22, 23]. Luangchana studied two CBCT systems and showed that the linear measurement data of CBCT were accurate enough [24]. The SINODE GALILEOS CBCT image used in this research has ideal clarity and accuracy. Before the operation, the robot manufacturer staff tested the CBCT accuracy of this research group, and its size measurement accuracy is less than 0.1 mm, which can meet the needs of the robot system used in this research. The robot software system can extract CBCT data and verify it, which can realize the implanting operation under the guidance of robot.

This study investigated the feasibility of robotic applications for clinical zygomatic implants based on model studies and the reported clinical case. In the model experiment, the micro-motion of anatomical structure, mouth opening and head is simulated, and the robot drilling process is realized under the condition closer to the actual clinical scene. The “follow me” function of the mechanical arm allows synchronous movement with the patient’s head (within 0.1 s). There was no significant difference between zygomatic implant implantation experimental group and alveolar bone implant implantation control group (*p* > 0.05). The mean values of the entry point error and the angle error of zygomatic implants were slightly less than in the study by Cao et al. [20], meanwhile, the mean values of the entry point error and the angle error of alveolar implants were slightly smaller than in the study of Bolding et al. [13]. The precision of the model experiment outcomes provided us with a numerical foundation and assurance of safety for undertaking clinical robotic zygomatic implant surgery. In the clinical case of this study, the mean deviations of the two zygomatic implants fell within the range of the results of the model experiment.

In the process of robot-assisted surgery, marking is very important for the registration of robot arms. The marker contains blocking points on a fixed substrate for repeated CBCT data fusion, and a calibration image board for accurate registration. The design of the marker should be stable, minimally invasive, not easy to deform, compact in size and easy to wear. Traditional markers are attached to the remaining teeth and cannot be fixed in edentulous patients, so they need to be replaced during bilateral surgery, which increases the inaccuracy of positioning and the operation time. In this study, the fixing base of the maker is designed in the shape of “human”, which makes it smaller and more suitable for the patient’s anatomical structure, so it is more stable and comfortable to wear (Fig. 4b). During the operation, screws are used to fix the base to the maxilla of the palate, where there are more bones to prevent interference from loose or missing teeth. The herringbone is extended to adapt to soft tissue and increase overall stability. In addition, the design of the marker allows it to be placed in the anterior region, bypassing the bilateral surgical regions, and allowing simultaneous surgery on both sides without replacing the marker. The arrangement of blocking points on the fixation base of the marker aided the integration of several CBCT data sets and decreased overlap errors in radiographs. The connector between the fixation base and the calibration image plate bypassed the anterior region and the upper lip to avoid distortion and extrusion which could increase the deviation.

The calibration image board on the mark of Remebot system is composed of black and white blocks, which can be reliably identified using ambient light recognition technology under natural lighting conditions. The calibration plate is positioned to avoid the operation area as much as possible and is not easily blocked by the operator. At the same time, the image data of the patient and the environment are collected, which can allow the progress of visual field image processing. The Yakebot system currently uses infrared recognition technology for calibration with a single wavelength of infrared light as a light source, which only acquires the position and image data of the marker in the field of view and no additional image data of the surrounding environment. The Yomi robotic system utilized a physical tracking system that was physically attached to the patient and the operating devices and could only give physical tactile guidance.

During the clinical robotic zygomatic implant surgery, flap and bilateral maxillary sinus lateral wall openings were performed first. This precaution was taken for two safety-related reasons: (1) the mucosa of maxillary sinus is peeled off after the valve is opened, which prevents the mucosa of maxillary sinus from being damaged during drilling and (2) the robot drilling process can be seen under direct vision, which provides additional patient protection. The traditional robot implantation surgery first fixes the marker, and then performs the implantation operation with or without valve opening. However, considering the potential interference of skin flap, peeling and other surgical operations on the stability or shape of the marker, in this clinical case, the marker is fixed and then registered with the mechanical arm to improve the accuracy. It is mentioned in the literature that the zygomatic implant site may pose a threat to the eyeball or lead to serious complications, such as sinus infection or skin penetration [25, 26]. Therefore, before implantation, intraoperative CBCT should be performed to reconfirm the safety and stability of the implantation site. The CBCT was taken using a low dosage of radiation, and with the maturation of robotic zygomatic implant technology, the examination might be shortened to 2 times (1 taken preoperatively with the marker and 1 taken postoperatively).

**Fig. 12:**
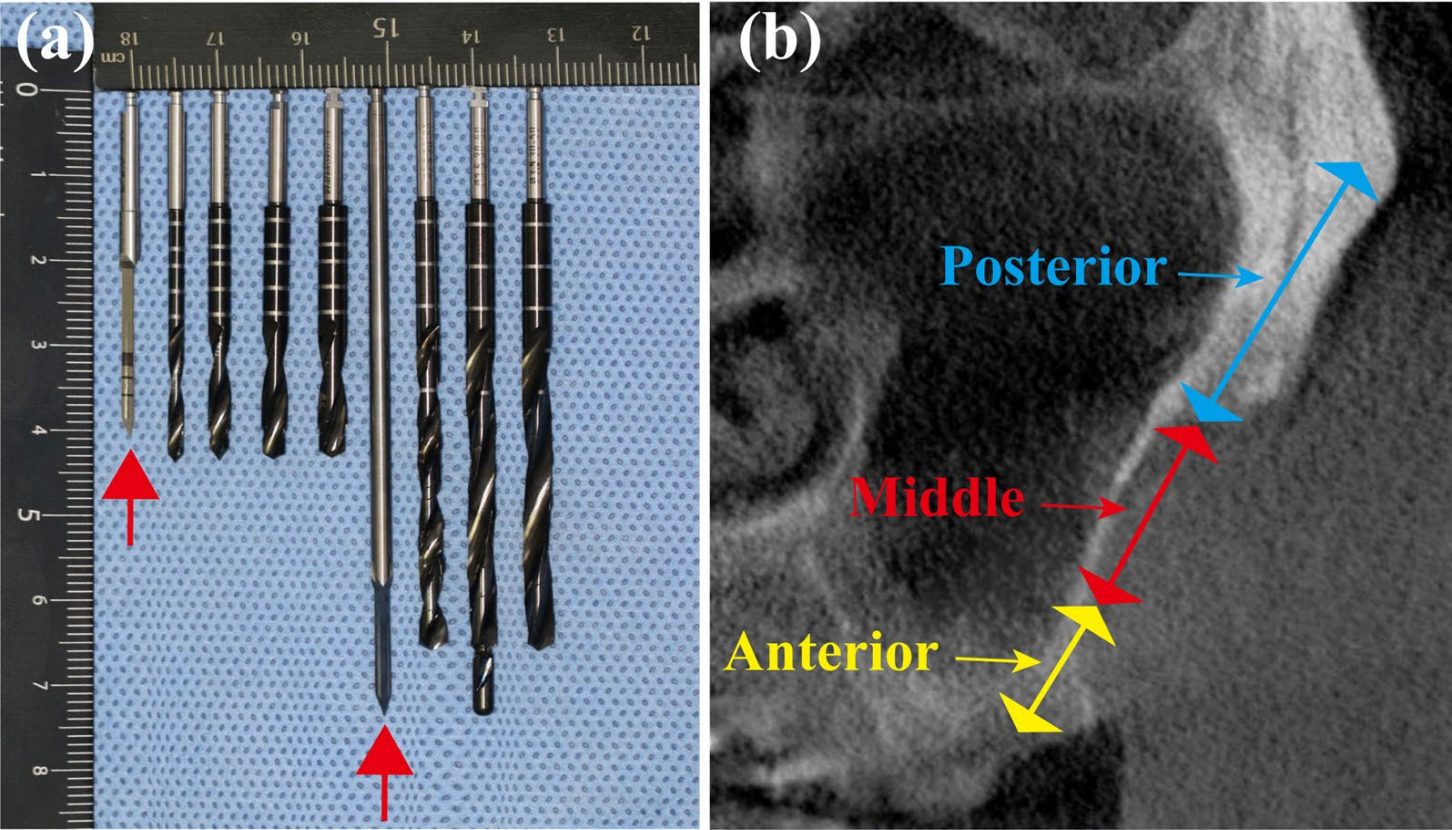
Custom-made surgical precision drills were used during robotic zygomatic preparation to prevent drill tip slippage and deflection and the sequence of the drills was optimized, as indicated by the red arrow (**a**). The site preparation procedure consisted of three phases including anterior preparation, middle preparation and posterior preparation for safety and accuracy of the robotic drilling process

To prevent drill tip slippage and deflection while drilling with extra-long drills, in this study custom-made surgical drills of trigonometric drill 2.0 × 33 mm and trigonometric drill 2.0 × 74.8 mm were developed, with sharp drill tips and no side slippage (Fig. 12a). The sequence of drills utilized in the robotic zygomatic site preparation process was optimized for considerable reasons. The drilling procedure consisted of three phases: pre-preparation, intermediate preparation and post-preparation (Fig. 12b). The anterior preparation is performed in the alveolar area. The precision drill 2.0 × 33 mm was used for anterior positioning, and then the twist drill 2.0 × 31 mm, and the twist step drill 2.4/2.8 × 31 mm, 2.8/3.2 × 31 mm, 3.2/3.6 × 31 mm were used to complete the front road preparation, so as to eliminate the front segment resistance. During the middle preparation, the drill needed to pass through the lateral wall area of the maxillary sinus, the long drill was previously more prone to slip and deflection [20]. In this case, the lateral wall opening was completed in advance, allowing the robotic drilling operation to go directly to the zygomatic region without interference from the lateral bone wall. The posterior preparation was performed in the zygomatic region. The trigonometric drill 2.0 × 74.8 mm entered the zygomatic area without obstruction and was precisely positioned. Then followed by twist drill 3.0 × 67.5 mm, pilot drill 3.5 × 75 mm, and twist drill 3.5 × 67.5 mm to complete the posterior segment preparation. The operation strategy of the robot in the cheekbone area is to enter 5 mm and retreat 3 mm, which is cyclic and provides enough water cooling during drilling to prevent the long drill from generating too much heat.

Because of the super-long drill bit (67.5 mm) required by the robot zygomatic implant, it is difficult to complete oral surgery in the mouth of ordinary patients. To solve this problem, on one hand, a customized metal handpiece holder for robotic zygomatic implant surgery was developed in this study (Fig. 5a), which was small and flexible in movement. On the other hand, preoperative planning placed the implant path relatively close to the premolar area, which, in conjunction with the large mouth opening of the patient in this study, allowed the robotic handpiece to enter and exit the mouth with the lengthy drill. The design of zygomatic implant placement is based on the shape or concavity of maxillary anterior wall and the vertical/horizontal bone absorption degree of alveolar bone. This case is close to ZAGA) II classification [27]. The design of the zygomatic implant site is reasonably close to the premolar area, and there is enough bone mass around it, which provides ideal biomechanical support, initial stability and withdrawal position. During the operation, it was noticed that the 100 mm drill was extremely difficult to enter and exit the internal space, even with the assistance of the upper lip and the lower lip. In this case, the loss of mandibular anterior teeth and a wide mouth of at least 4.5 cm may be beneficial to the successful operation. However, for patients with insufficient mouth opening, which is relatively prevalent, especially in elder patients, the use of robotic systems in zygomatic implant placement may be hindered. Further studies need to be conducted and more modifications need to be made to optimize the autonomous robotic surgery system. The strategies for robotic zygomatic implants, the operation procedures, and the hardware and software of the robotic system urge further improvement.

In a word, the optimal design of markers, the treatment of surgical area with advanced flaps and maxillary sinus side wall openings, and the improvement and optimization of triangular drills and hand-held brackets for robot cheekbone implantation are all helpful to reduce robot implantation errors. According to the surgical procedure and method developed in this study, the accuracy of the clinical application of a robotic zygomatic implant was reliable and the overall deviation was small with no resistance of the lateral maxillary sinus wall. With the continual advancement and optimization of digital technology, the use of the robot will be able to provide a higher level of assurance for the precision and safety of robotic zygomatic implant surgery.

## Conclusion

The accuracy of in vitro model zygomatic implant placement experiment realized by Remebot robot surgery system is equivalent to that of ordinary implant placement. The successful application of autonomous robot system in the current zygomatic cases proves the feasibility of robot zygomatic surgery for immediate maxillary implant loading. With the continuous improvement and optimization of digital technology, the application of autonomous implanted surgical robot will realize accurate, safe and minimally invasive patient-specific surgery. Further clinical trials are needed to provide high-quality clinical evidence.

## Data Availability

The data set supporting the conclusions of this article are included within the article. Further data sets are available from the corresponding author upon reasonable request.
