# Correction: Autonomous robotic surgery for zygomatic implant placement and immediately loaded implant-supported full-arch prosthesis: a preliminary research

**DOI:** 10.1186/s40729-024-00553-y

**Published:** 2024-10-07

**Authors:** Changjian Li, Menglin Wang, Huanze Deng, Shumao Li, Xinyu Fang, Yijie Liang, Xihua Ma, Yue Zhang, Yanfeng Li

**Affiliations:** 1 Department of Stomatology, The Fourth Medical Centre, Chinese PLA General Hospital, Beijing, 100048 China; 2 Medical School of Chinese PLA, Beijing, China; 3 Department of Orthopedics, The Fourth Medical Center of PLA General Hospital, Beijing, 100048 China

**Correction: International Journal of Implant Dentistry (2023) 9:12**

10.1186/s40729-023-00474-2

In the original publication of this article there was an incorrect, non-functional contact address listed. The incorrect and correct information is listed in this correction article. The original article has been updated.

**Incorrect**

494955789@qq.com

**Correct**

949427779@qq.com

